# Metal-rich stars are less suitable for the evolution of life on their planets

**DOI:** 10.1038/s41467-023-37195-4

**Published:** 2023-04-18

**Authors:** Anna V. Shapiro, Christoph Brühl, Klaus Klingmüller, Benedikt Steil, Alexander I. Shapiro, Veronika Witzke, Nadiia Kostogryz, Laurent Gizon, Sami K. Solanki, Jos Lelieveld

**Affiliations:** 1 Max Planck Institute for Solar System Research, Göttingen, Germany; 2 Max Planck Institute for Chemistry, Mainz, Germany; 3 Institute for Astrophysics, Georg-August-Universität Göttingen, Göttingen, Germany; 4 Center for Space Science, NYUAD Institute, New York University Abu Dhabi, Abu Dhabi, UAE; 5 School of Space Research, Kyung Hee University, Yongin, Republic of Korea; 6 The Cyprus Institute, Climate and Atmosphere Research Center, Nicosia, Cyprus

**Keywords:** Exoplanets, Stars, Astrobiology

## Abstract

Atmospheric ozone and oxygen protect the terrestrial biosphere against harmful ultraviolet (UV) radiation. Here, we model atmospheres of Earth-like planets hosted by stars with near-solar effective temperatures (5300 to 6300 K) and a broad range of metallicities covering known exoplanet host stars. We show that paradoxically, although metal-rich stars emit substantially less ultraviolet radiation than metal-poor stars, the surface of their planets is exposed to more intense ultraviolet radiation. For the stellar types considered, metallicity has a larger impact than stellar temperature. During the evolution of the universe, newly formed stars have progressively become more metal-rich, exposing organisms to increasingly intense ultraviolet radiation. Our findings imply that planets hosted by stars with low metallicity are the best targets to search for complex life on land.

## Abstract (web summary)

Low stellar ultraviolet (UV) radiation leads to low ozone abundances, therefore, less planetary UV protection. Here, the authors show that planets in the habitable zones of metal-poor stars, despite their higher UV radiation than metal-rich stars, are the best targets for search for life.

## Introduction

Complex, multicellular life on land requires oxygen (O_2_) from which ozone (O_3_) forms^1^, leading to a tolerable ultraviolet radiation (UV) level at the surface for its development and evolution^2–4^. Stellar emission and planetary UV protection depend on the effective temperature of the host star^5–7^. While for a young planet UV exposure can be essential for abiogenesis^8–11^, high levels of UV trigger genomic damage and are a threat to all life forms^12–14^. In the Sun-Earth system the UV-C (202 to 230 nm, wavelengths potentially reaching the surface in oxygenated atmospheres) and UV-B (280 to 315 nm) fluxes at 1 Astronomical Unit (au) from the Sun are about 0.76 W/m^2^ and 20 W/m^2^, respectively^15^. This is well above the maximum tolerable level for terrestrial life. Land-based life has nevertheless evolved on Earth through oxygen enrichment of the atmosphere that blocks most of the UV radiation. While UV-C is largely absorbed by O_2_ molecules in the upper atmosphere, UV-B is absorbed by the ozone layer in the middle atmosphere.

The O_3_ concentration is regulated by a photochemical cycle of O_2_ and O_3_ dissociation by solar UV radiation in the Herzberg continuum (200 to 242 nm) and Hartley band (200 to 320 nm), respectively^1^. Only the longer wavelengths (260 to 320 nm) of the Hartley band are relevant for the O_3_ column density burden because radiation at shorter wavelengths is mostly absorbed by O_2_. Ozone absorption in the band center (260 nm) is so strong that radiation can hardly penetrate to the middle atmosphere where O_3_ concentrations are highest (Supplementary Fig. 1 and 3). Hence, the O_3_ concentration depends on the balance between stellar irradiance in the 200 to 242 nm and 260 to 320 nm spectral bands, which govern the production and destruction of O_3_, respectively (hereafter we refer to the net photochemical effect). Consequently, the UV-protection provided by the planetary atmosphere depends on the spectral distribution of the stellar radiation^5,7,16^.

The stellar radiative spectrum, in turn, depends on the effective temperature, T_eff_, and metallicity, [Fe/H], that represents the abundance of elements heavier than hydrogen and helium in a star (see Eq. 4 in methods section Stellar spectra). The dependence of the radiative conditions at the planetary surface on the stellar effective temperature has been studied previously. For example, it was shown that with increasing effective temperature the planetary surface UV above 290 nm also increases, but the radiative transfer at shorter wavelengths is non-monotonous due to spectrally dependent photochemical effects^5,7^.

Here, we investigate the dependence of planetary surface UV on the atmospheric O_2_ concentration and stellar metallicity for stars of three spectral types: G2V (T_eff_ =5800 K, representing solar case), G5V (T_eff_ =5300 K), and F7V (T_eff_ =6300 K). We note that the G5V and F7V classes encompass roughly 50% of the presently known planetary hosts. We account for a range of metallicity values between −1 and 0.9 dex which covers most planetary hosts. We first consider the development of the Sun-Earth system in the past 0.5 billion years as a model for planets and their host stars, during which the atmosphere was oxygenated and complex life on land evolved. We then study the dependence of surface UV irradiation on the atmospheric O_2_ content and stellar metallicity. We show that the development of complex life on planets in the habitable zone can be sustained from a few percent of O_2_ upward, being robust for a large range of stellar characteristics and against major extraterrestrial cataclysms.

## Results

**Fig. 1:**
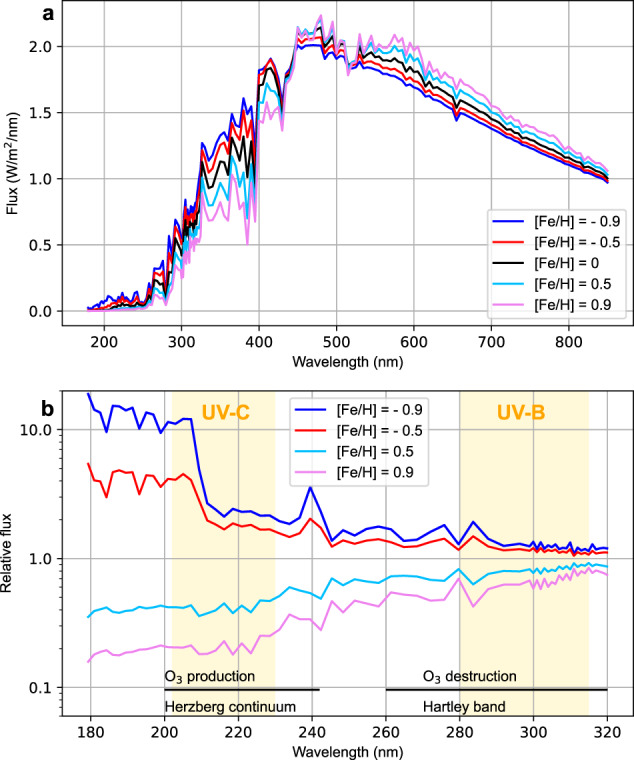
Stellar radiative spectra. **a** Stellar spectra calculated for different metallicities [Fe/H] and a solar effective temperature T_eff_ of 5800 K. The calculations were performed for a spectral range of 170 to 850 nm and the total flux normalized to the solar constant. **b** The same as **a** but the flux is shown relative to the solar flux ([Fe/H] = 0). Radiation in the 200 to 242 and 260 to 320 nm intervals participates in O_3_ production and destruction, respectively. These spectral ranges are defined by black solid lines. The yellow shaded areas show the spectral ranges of the UV-C reaching the lower atmosphere (202 to 230 nm, left) and UV-B (280 to 315 nm, right). Source data are provided as a Source Data file.

Figure 1 presents stellar spectra calculated for different metallicity values using the recent MPS-ATLAS code^17^. The UV flux drops substantially with increasing metallicity, creating seemingly more favourable conditions for life^18^. However, Fig. 1b shows that metallicity affects radiation in the O_3_-producing Herzberg continuum much more strongly than in the O_3_-destroying Hartley band. Thus, the net photochemical effect leads to a decrease of O_3_ with metallicity, making the assessment of the UV conditions and potential habitability at the planetary surface less straightforward, which was hitherto not accounted for.

We consider hypothetical Earth-twin planets (with an N_2_/O_2_ atmosphere, water and terrestrial mass) in the habitable zones of stars with T_eff_ and [Fe/H] in ranges introduced above. The O_3_ concentration in planetary atmospheres and the associated surface UV levels were computed with a coupled photochemical radiative-convective model^19^. The model has been designed and updated for accurate calculations of atmospheric O_3_ chemistry. It avoids assumptions of fixed boundary conditions, e.g. of methane (CH_4_) concentrations, and interactively computes the oxidation capacity of the atmosphere for different UV conditions, which is relevant for the removal of hazardous and greenhouse gases. The radiative scheme has a high spectral resolution, which appears to be critical for studying the impact of stellar radiation on planetary atmospheres^1^. The description of the planetary atmospheric model and our calculations of the input stellar spectra are presented in the methods sections Atmospheric model and Stellar spectra. We consider three key factors for a life-supporting UV environment on surfaces of planets: (1) UV-C level (relevant in low oxygen atmospheres); (2) the oxidation capacity of the lower atmosphere (regulated by UV radiation); (3) UV-B level (e.g., regarding DNA damage^2,3^). Further, the atmospheric protection mechanism should be able to tolerate large disturbances, e.g. by volcanic eruptions and supernova explosions (which can destroy O_3_^20,21^) as well as changes of the activity of the host star^22^ which affects its radiation spectrum^23^ (see methods sections Ozone and UV-B response to perturbations and Stellar spectra).

### The Sun-Earth evolution

A quantitative assessment of the biological impact of these factors is challenging because additional protection (e.g. water bodies, shadowing by rocks, pigment formation) and biological repair mechanisms are not known. Therefore we considered the Earth-Sun system as a paradigm to guide the interpretation of our results for other systems. We first investigated how life on land of our planet steered through the conditions mentioned above and then how these conditions are affected by the effective temperature and metallicity of the host star.

**Fig. 2:**
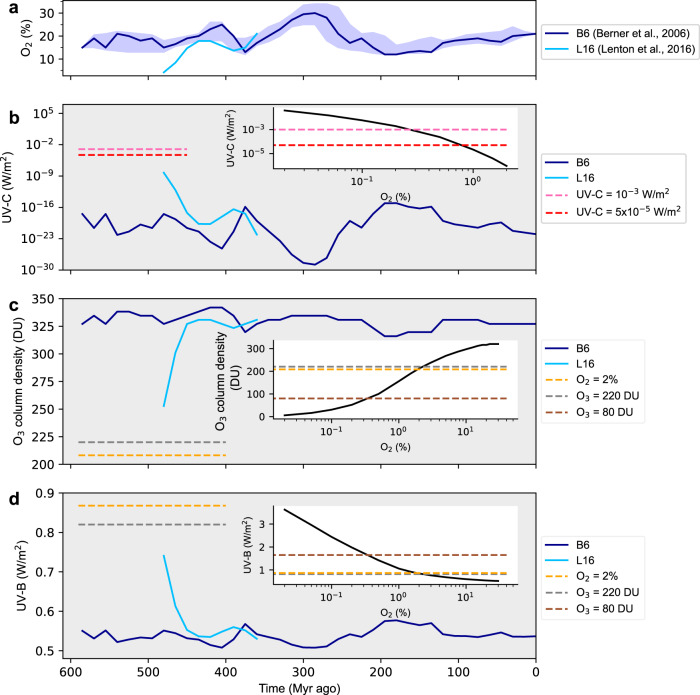
Evolution of atmospheric O_2_, O_3_ column density and surface UV. **a** O_2_ volume fraction of the Earth’s atmosphere during the past 600 million years according to Berner et al., 2006^24^ (B6, blue) and Lenton et al.^27^ (L16, light blue). The blue shaded area indicates the uncertainty range of the B6 data. **b**–**d** Surface UV-C (202 to 230 nm) **b**, O_3_ column density **c** and surface UV-B (280 to 315 nm) **d** (1 DU = 2.687 × 10^20^ molecules/m^2^). The blue and green curves are calculated with B6 and L16 data, respectively. The inserted plots show the dependencies of UV-C **b**, O_3_ column density **c** and UV-B **d** on the O_2_ content. **c**, **d** The level of O_3_ corresponding to 2% of O_2_ is shown by the yellow dashed line. The O_3_ level of 220 and 80 DU are indicated by the grey and brown dashed lines, respectively. The UV-C levels of 10^−3^ and 5×10^−5^ W/m^2^ are shown by the pink and red dashed lines in **b**. Source data are provided as a Source Data file.

Figure 2 illustrates the development of the Earth’s atmosphere and surface UV fluxes over the last 600 Myr (million years before present). The geological isotope records indicate that the level of atmospheric O_2_ (and CO_2_, see Supplementary Table 1) went through substantial fluctuations^24–27^ (Fig. 2a). The largest change in O_2_, known as the Paleozoic oxygenation event, happened around 470 Myr^28^ when Earth’s atmosphere went from almost anoxic to oxygenated conditions. While this event is absent in the reconstruction of Berner et al.^24^ (B6, blue curve in Fig. 2a) data (probably because of simplified assumptions about sulfur geochemistry^29^), it is evident in the more recent reconstruction of Lenton et al., 2016^27^ (L16, light blue curve in Fig. 2a).

Before the Paleozoic oxygenation O_2_ was mainly produced by aquatic photosynthesis in UV tolerant cyanobacteria and algae^26,30,31^ which could only provide a limited amount of O_2_: while L16 indicates 4% of O_2_ shortly before the event, other studies point to lower values of 2%^32^ or even 0.2%^26,33^. It is understood that the Paleozoic oxygenation event was caused by the advent of the earliest land plants^27,29^. This transition likely represents an important bottleneck: effective O_2_ release to the atmosphere is not possible without land plants, which in turn are susceptible to UV radiation^33,34^ and can only appear when the O_2_ concentration is sufficiently high to create a protective ozone layer. We have modelled the history of the Earth’s atmosphere to calculate the O_2_ and O_3_ concentrations needed to overcome the oxygenation bottleneck on Earth and simultaneously establish quantitative criteria on the non-hostile level of UV irradiance.

**Fig. 3:**
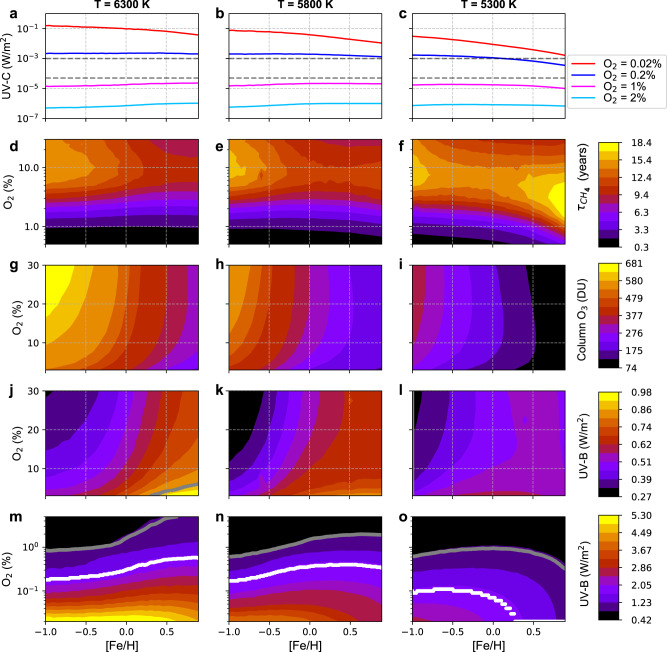
Metallicity impacts on the atmosphere. **a**–**c** The change of surface UV-C (202 to 230 nm, W/m^2^) with [Fe/H] for O_2_ levels of 2% (light blue), 1% (magenta), 0.2% (blue) and 0.02% (red). The dashed lines indicate the UV-C levels of 5 × 10^−5^ and 10^−3 ^W/m^2^. **d**–**f** Impact of O_2_ content and [Fe/H] on CH_4_ lifetime. **g**–**l** Dependencies of O_3_ column density (**g**–**i** DU) and surface UV-B (**j**–**l** W/m^2^) on O_2_ content and stellar metallicity [Fe/H]. **m**–**o** The same as j-l but for an O_2_ content less than 5%. **a**–**o** The calculations were performed for stellar *T*_eff_ of 6300 K (left), 5800 K (center) and 5300 K (right). The grey and white curves (**j**–**o**) represent surface UV-B of 0.82 W/m^2^ (220 DU, ozone hole definition) and 1.65 W/m^2^ (80 DU, extreme in ozone hole), respectively. Source data are provided as a Source Data file.

Here we consider the evolution of UV-C and UV-B fluxes at the surface. While UV-C is particularly harmful to living cells due to the highly energetic photons, Fig. 2b shows that a flux of 10^−3^ Wm^−2^, corresponding to a tolerable annual dose in the order of 10^4 ^J m^−2^ ^35^, is achieved already at 0.3% of O_2_. This is below the O_2_ level after the Paleozoic oxygenation event. It is also below or comparable to available estimates for the O_2_ levels preceding the event^27,32,33^. All in all, we do not expect the UV-C irradiance to pose a critical threat to the advent of land plants directly. Long CH_4_ lifetimes resulting from our calculations, being a metric of the atmospheric oxidation capacity, indicate that the low UV-C values allow a mildly oxidative environment supporting the removal of hazardous gases in the lower atmosphere while not exposing organisms to harmful oxidant levels. It means that the atmosphere is not chemically aggressive, i.e. hostile to the organic molecules of living cells at UV-C < 5 × 10^−5^ W/m^2^ or O_2_ > 0.8% (Figs. 2b and 3e and Supplementary Fig. 5k). Under these more moderately oxidative conditions, the CH_4_ lifetime exceeds about a year and thus does not drop below about a tenth of that in the atmosphere of today.

Since UV-B is mainly absorbed by O_3_, the surface UV-B level must be calculated together with the atmospheric chemistry and O_3_ concentration. We show the results of such calculations in Fig. 2c, d. Previous work^5^ has shown that O_3_ column density is resilient even to strong changes of O_2_ (Fig. 2c). Our study reveals that the Paleozoic oxygenation event, which corresponds to a change in O_2_ by a factor of 4.5 (L16 data), resulted in a mere 30% change of O_3_ column density (from 252 to 330 Dobson Units, DU) leading to a moderate UV-B response (from 0.75 to 0.55 W/m^2^, Fig. 2d). The low sensitivity of O_3_ column density and, consequently of UV-B, to changes in O_2_ is explained by the vertical adjustment of the ozone layer. Smaller O_2_ amounts cause the ozone layer to form in lower and denser atmospheric layers where more O_2_ is available for O_3_ production (Supplementary Fig. 3a, b), leaving the total O_3_ column density only weakly affected^36^.

The total O_3_ column density of 252 DU (Fig. 2c) calculated for the conditions shortly before the Paleozoic oxygenation event (L16 data) is well above the 220 DU (grey dashed line in Fig. 2c) that define the recent ozone hole over Antarctica^21^ and can be considered tolerable by the vegetation. Since the O_2_ concentration before the Paleozoic oxygenation event is rather uncertain we consider even lower oxygen levels. Lowering O_2_ to 2% results in O_3_ decrease to 205 DU (orange dashed line in Fig. 2c) and a UV-B increase to 0.87 W/m^2^ (Fig. 2d). Though this amount of O_3_ qualifies as an ozone hole, it is routinely measured over the Antarctic coastline^37^ and sometimes over southern Argentina^38^ or northern Europe where land plants are nevertheless abundant. Thus, also this level of UV-B does not pose a lethal threat to the terrestrial biosphere. The O_3_ column density and UV-B modelled for O_2_ below 2% are presented in the inserts of Fig. 2c, d. The O_3_ column density of 80 DU (brown dashed line in Fig. 2c) that corresponds to O_2_ of 0.3% is close to the lowest value measured over Antarctica in spring (September 30, 1994, with 73 DU) which was tolerable only due to the large solar zenith angle near the pole and the limited time period. Here, we consider the corresponding UV-B level of 1.65 W/m^2^ (Fig. 2d) as the highest value that is known to be survivable by land plants on present Earth.

Our calculations thus show that the dependence of the surface UV-B fluxes on the O_2_ amount is limited. We consider this to be a key factor, together with the atmospheric oxidation capacity, for allowing land plants on Earth, which might similarly apply to habitable planets orbiting other stars. Furthermore, during the past 470 million years the dependence of the Earth’s surface UV-B fluxes on the O_2_ amount has been limited because the O_2_ mixing ratio was well above 1%. Together with the atmospheric oxidation capacity, we consider this crucial for land plants on Earth, which might similarly apply to habitable exoplanets.

### Impacts of stellar properties

The UV emission by a star strongly depends on fundamental stellar parameters. It increases with the stellar effective temperature T_eff_^5–7^ (Supplementary Fig. 2) and decreases with the metallicity [Fe/H] (Fig. 1). We investigated how these parameters affect the correspondence between O_2_ in the planetary atmosphere and the surface UV thresholds indicated above. Note that O_2_ in exoplanetary atmospheres may also be provided by abiotic sources^39,40^. Figure 3a–c shows that for 1% of O_2_ the surface UV-C flux is lower than the level of 5 × 10^−5^ W/m^2^ for all stellar T_eff_ and [Fe/H] values. We find that for 0.2% or more O_2_ the higher UV-C fluxes of metal-poor stars are compensated by higher O_3_ concentrations resulting in almost metallicity-independent UV-C at the planetary surface (Fig. 3a–c and Supplementary Fig. 4). This is remarkable given the strong anti-correlation of stellar UV-C emission fluxes and metallicity (Fig. 1b). Interestingly, the UV-C level also marginally depends on stellar T_eff_. Thus, the O_2_ level that provides UV-C protection is very similar for a large range of stellar parameters.

In the case of very low O_2_ (< 0.7%), UV-C photolysis of H_2_O controls the formation of oxidants like excited O atoms and hydroxyl (OH) radicals (the latter known as the detergent of the atmosphere), which act as sinks for CH_4_ and other molecules released from geochemical processes. Consequently the lifetime of CH_4_ (τ_CH4_), which signifies the oxidation capacity of the atmosphere (Fig. 3d–f), is very short (below 1 year), which likely makes the planetary environment harmful for life on land. For O_2_ in excess of about 0.7% the OH concentration (Supplementary Fig. 5j–l) and τ_CH4_ are controlled by UV-B photolysis of O_3_, modulated by ambient concentrations of H_2_O, NO_x_ (mostly NO, NO_2,_) and CO. While surface O_3_ for O_2_ levels between 0.7 and 2% strongly depends on T_eff_ and metallicity (Supplementary Fig. 5d–f), UV-B in the lower planetary atmosphere responds much less sensitively (Fig. 3m–o), and so does τ_CH4_ (Fig. 3d–f). Above 2-3% O_2_, the atmospheric oxidation capacity is buffered, hence stable and benign to life, maintaining a τ_CH4_ within 8-18 years throughout the range of [Fe/H] and T_eff_. For details about the temperature and atmospheric composition, see methods section Atmospheric model and Supplementary Fig. 5.

The effects of the stellar temperature T_eff_ and metallicity [Fe/H] on UV are stronger in the Herzberg continuum, driving the chemical O_3_ production, than in the Hartley band which drives O_3_ destruction (see Fig. 1 for the [Fe/H] effect and Supplementary Fig. 2 for the T_eff_ effect). As a result, the net photochemical effect leads to the increase of the O_3_ column density with T_eff_ and a decrease with [Fe/H] (Fig. 3g–i). Thus, changes in UV irradiance at the planetary surface can respond in the opposite direction to those of the stellar UV radiance. The net photochemical effect is not sufficiently strong to reverse the UV-B surface flux dependence on T_eff_ for all metallicities and oxygen levels (Fig. 3j–l, Supplementary Fig. 2), but it generally reverses the dependence on [Fe/H] leading to the paradoxical anti-correlation of the surface and space UV-B fluxes (Figs. 1 and 3j–l). The increase of the surface UV-B with [Fe/H] is especially strong for T_eff_ values of 5800 and 6300 K but it is less pronounced for 5300 K stars (where surface UV-B starts to decrease with [Fe/H] for low O_2_ levels, see Fig. 3o).

The white lines in Fig. 3m, n indicate that for the T_eff_ values of 5800 and 6300 K approximately two times more O_2_ is needed to attain the extreme ozone hole conditions (i.e. UV-B flux of 1.65 W/m^2^) for [Fe/H] = 0.9 than for [Fe/H] = −1. The oxygen concentration required to arrive at lower UV-B levels such as ozone hole conditions (0.82 W/m^2^, black lines in Fig. 3k–o) depends even more strongly on the metallicity for 5800 K and 6300 K stars. For example, for the effective temperature of the Sun the required O_2_ concentration increases from 0.6% at [Fe/H] = −1 to about 2% for [Fe/H] > 0.5. The 5300 K stars do not show such a dependence. For example, the O_2_ value needed for the UV-B flux of 0.82 W/m^2^ marginally increases from an [Fe/H] of −1 to ≈ 0 but decreases for higher [Fe/H] (see black line in Fig. 3o).

Interestingly, the surface UV-B fluxes corresponding to atmospheres with an O_2_ level higher than 3% monotonically increase with [Fe/H] for all three T_eff_ values considered in this study (Fig. 3j, k, l, with the exception of [Fe/H] ≥ 0.8 and T_eff_ = 5300 K, see Fig. 3l). For example, for the T_eff_ of the Sun the increase of [Fe/H] from −1 to 0.9 doubles the surface UV-B flux. While surface UV-B fluxes on planets with oxygenated atmospheres may not pose a fatal threat for life on land, their increase with [Fe/H] could negatively affect the evolution of life, especially for planets with low O_2_ atmospheres, orbiting 5800−6300 K, high-metallicity stars. Since supernovae continuously enrich the Universe with heavy elements over time, stars that form later increasingly contain heavy elements and their planets provide less favourable UV conditions for vegetation and the advancement of complex land life.

While a high stellar metallicity causes UV-B stress for developed life in oxygenated atmospheres, it might be accompanied with faint UV-C radiation levels, being insufficient for the photochemical formation of essential macromolecular building blocks of life in early anoxic atmospheres. For example, it was recently estimated^10^ that the average UV actinic flux between 200 and 280 nm should exceed about 6 × 10^9^ cm^−2^ s^−1^ Å^−1^ at the planetary surface to allow abiogenesis. Supplementary Figs. 6 and 7 show the dependence of the surface actinic flux on the effective temperature T_eff_ and metallicity [Fe/H] for the 80%-nitrogen and 20%-carbon dioxide atmosphere calculated using the atmospheric transmission dependence on wavelength adopted from Rimmer et al.^10^ (we note that in contrast to the oxygenated atmospheres the opacity in anoxic atmospheres is not affected by the net photochemical effect and, thus, is not expected to depend on the stellar spectrum). The actinic flux decreases for cooler and for metal-rich stars, making them less life-friendly. In particular, the actinic flux drops below the Rimmer et al.^10^ estimate for 5300 K metal-rich stars.

## Discussion

A key factor for the development of land life is the stability of the atmospheric UV shielding in response to major disturbances that are likely to happen on geological time scales. We performed sensitivity simulations to study the atmospheric effects of potentially catastrophic events such as sudden increases of stellar activity, supernovae and volcanic eruptions. Our model results show that independent of the stellar metallicity such cataclysms do not pose planetary scale, existential threats to life (see methods section Ozone and UV-B response to perturbations and Supplementary Figs. 8–10). While the largest supervolcanoes on Earth have occasionally caused major species extinctions, there are no known examples of such events that have annihilated life^41^.

Our results show that from a few percent of O_2_ upward and for a variety of stellar properties the surface UV exposure on Earth-like planets in habitable zones is likely to sustain the development of land plants that are essential for the further evolution of complex life. It includes a stable oxidation capacity of the atmosphere, which controls greenhouse gases such as CH_4_, contributing to favourable temperature conditions, while removing hazardous gases that would otherwise reach toxic levels. Paradoxically, whereas stars with higher metallicity, which have appeared later in the history of the Universe, emit less UV radiation, in oxygenated planetary atmospheres the associated stellar radiative spectrum allows less O_3_ formation, which enhances UV penetration, making the conditions on planets orbiting these stars less friendly for the biosphere on land (except for 5300 K metal-rich stars and low O_2_ atmospheres). The relatively low UV emission from the high-metallicity stars can also be a hurdle for the origin of first life on planets with anoxic atmospheres.

We thus find that the surface of planets orbiting metal-rich stars is exposed to more intense UV radiation than the surface of planets orbiting metal-poor stars. Therefore planets in the habitable zones of stars with low metallicity are the best targets to search for complex life on land. For the stellar types considered, metallicity has a larger impact on the surface UV than the stellar temperature. The atmospheric oxidation (cleaning) capacity is found to be stable and life-supporting, almost independent of stellar metallicity at an oxygen volume fraction above 1%.

The new generation of radial velocity (RV) spectrometers will be able to measure stellar reflex motion with a precision of 10 cm s^−1^ ^42^ which suffices to discover Earth-like planets in the habitable zones of Sun-like stars. Detecting Earth-like planets orbiting Sun-like stars is also the main objective of the upcoming PLAnetary Transits and Oscillations (PLATO)^43^ of stars space telescope. Our results indicate that to maximise the likelihood of finding signatures of life, planets hosted by low-metallicity stars discovered by these instruments should be priority targets of the follow-up observations with future telescopes^44^.

The recently commissioned James Webb Space Telescope (JWST)^45^ targets atmospheres of rocky planets around red dwarfs, i.e. stars significantly cooler and smaller than the Sun (since the signal from planets orbiting Sun-like stars is too low to be detected). While planets orbiting red dwarfs are not within the parameter range considered here, one future application of our model will be to simulate the spectral fingerprints of planetary atmospheres^5,6,46–49^ observable by JWST as well as anticipated ground-based facilities (like a 2040 s Large Infrared/Optical/Ultraviolet Space Telescope^50^).

## Methods

### Atmospheric model

We applied an updated, global one-dimensional radiative-convective model of a primarily nitrogen/oxygen atmosphere with interactive chemistry^19^ for simulations of a wide range of O_2_ levels and UV-spectra dependent on stellar properties and chemical perturbations. Photolysis rates are calculated with fine spectral resolution (176 wavelength intervals, delta-two-stream method) using equinox conditions and 6 zenith angles for the calculation of daytime average radiation fluxes, taking into account scattering and absorption interactively. Short-lived chemical species are assumed to be in local steady state, while longer-lived ones and chemical families are vertically redistributed by an eddy transport parameterisation. O_3_ is part of the odd oxygen-family which also contains atomic oxygen in the ground and excited states and radicals of the rate limiting reactions in catalytic destruction cycles of the form

1 $${{{{{{\rm{O}}}}}}}_{3}+h\nu \to {{{{{{\rm{O}}}}}}}_{2}+{{{{{\rm{O}}}}}}$$

2 $${{{{{\rm{X}}}}}}+{{{{{{\rm{O}}}}}}}_{3}\to {{{{{\rm{XO}}}}}}+{{{{{{\rm{O}}}}}}}_{2}$$

3 $${{{{{\rm{XO}}}}}}+{{{{{\rm{O}}}}}}\to {{{{{\rm{X}}}}}}+{{{{{{\rm{O}}}}}}}_{2}$$

X can be NO, OH or halogen atoms. NO and OH are produced in the middle atmosphere from reaction of an excited O-atom from O_3_ photolysis in the UV-B with N_2_O and H_2_O, respectively. Destruction of odd oxygen also occurs via the Chapman-reaction, which slows down with lower temperatures, e.g. due to radiative cooling by CO_2_:

4 $${{{{{\rm{O}}}}}}+{{{{{{\rm{O}}}}}}}_{3}\to 2{{{{{{\rm{O}}}}}}}_{2}$$

O_3_ production is governed by the photolysis of molecular oxygen by UV-radiation with wavelengths shorter than 242 nm, followed by

5 $${{{{{\rm{O}}}}}}+{{{{{{\rm{O}}}}}}}_{2}+{{{{{\rm{M}}}}}}\to {{{{{{\rm{O}}}}}}}_{3}+{{{{{\rm{M}}}}}}$$

with M an arbitrary atmospheric molecule (e.g. N_2_, O_2_).

The greenhouse gases H_2_O, CO_2_, CH_4_, O_3_, and N_2_O, predominant on Earth, are included in the near and terrestrial infrared radiative transfer calculations. Short-wave radiation reflected to space is determined from the high spectral resolution module used for the photolysis calculations. In the radiation calculations a climatological cloud cover is included and the surface albedo is fixed at conditions on Earth. The surface temperature (Supplementary Fig. 5) is calculated from the radiation budget at the top of the atmosphere, considering the dependence of the near-infrared part of the stellar spectrum on the effective temperature of the star. It is assumed that the total incoming radiative energy flux at the top of the planetary atmosphere is the same as the solar constant for present day Earth. Lower atmospheric temperatures are calculated from an approximately moist adiabatic lapse rate while middle atmospheric temperatures result from radiative equilibrium. The water vapor feedback for greenhouse warming, i.e. through evaporation from the surface, is included by assuming a fixed relative humidity profile in the lower atmosphere.

The surface boundary conditions for O_2_ and CO_2_ on paleo-Earth are established following available geological reconstructions^24,25,27^, see Supplementary Table 1. For the atmospheres of exoplanets CO_2_ volume mixing ratios were fixed to preindustrial (Holocene) conditions on Earth.

Because of the paucity of geological data we used pre-industrial lower boundary conditions for CH_4_, N_2_O, NO_*x*_, and CO fluxes (230 Tg/yr, 12 Tg/yr, 30 Tg/yr NO_2_, 1500 Tg/yr, respectively, CO fluxes include oxidation products of volatile organic compounds from land plants) in all simulations. Typical results for the surface are shown in Supplementary Fig. 5. The oxidation of organic substances such as CH_4_ proceeds via

6 $${{{{{\rm{RH}}}}}}+{{{{{\rm{OH}}}}}}\to {{{{{\rm{R}}}}}}+{{{{{{\rm{H}}}}}}}_{2}{{{{{\rm{O}}}}}}$$

where R = CH_3_ in the case of methane. In the presence of NO_*x*_ the further oxidation of R leads to a recycling of OH. The NO_*x*_ originates from soil emissions (bacteria), lightning and N_2_O breakdown in the middle atmosphere. Primary production of OH proceeds by reaction of an excited oxygen atom from the photolysis of O_3_ with H_2_O, and in the case of very low oxygen content by the UV-C photolysis of H_2_O (Supplementary Fig. 5). The altitude of the maximum O_3_ concentration, which decreases with stellar metallicity, shifts downward following the oxygen mixing ratio (Supplementary Fig. 3).

### Ozone and UV-B response to perturbations

In Supplementary Fig. 8b–e we show the O_3_ column density and surface UV-B levels calculated for the reference simulation, (black), conditions corresponding to a sudden increase of solar magnetic activity (yellow), a supernova explosion (magenta), and a major volcanic eruption (green), to test the stability of atmospheric conditions and compare with previous work. The reference simulation represents the pre-industrial Holocene state of the Earth’s atmosphere (i.e. surface fluxes of CH_4_, N_2_O, NO_x_ and CO are set to pre-industrial levels) under a stellar radiation intensity according to the minimum of solar cycle 22.

The high activity simulations are forced by the solar spectrum calculated for the Sun with an S-index value of 0.25, which corresponds to a magnetic activity level five times higher than during the maximum of solar cycle 22. This is motivated by the recent analysis of data from the Kepler space telescope^51^ and Gaia space observatory^52^ which hinted that the Sun might go through occasional epochs of high magnetic activity^22,53^. The intensification of stellar activity results in an increase of UV emission (Supplementary Fig. 11) that is stronger for radiation with shorter wavelength^23^. Consequently, the O_3_ production rate increases more strongly than the destruction rate resulting in an increase of the total O_3_ column density (Supplementary Fig. 8b). This result agrees with previous studies of the O_3_ response to the solar activity cycle^54,2^ which also reported a positive correlation between O_3_ column density and solar magnetic activity. The increase in O_3_ enhances the protection from UV-B which overcompensates the increase of the solar UV-B for an O_2_ content larger than 3% and damps it for smaller O_2_ amounts. Consequently, the increase of stellar magnetic activity does not pose a serious threat to the biosphere in terms of UV-B exposure.

Another potential perturbation is a supernova explosion of a neighbouring star^14,55^ that increases the flux of charged particles entering the planetary atmosphere^20^. On Earth, most particles are deflected by the heliospheric and geo-magnetic fields, but a fraction enters the atmosphere and produces NO_*x*_ from molecular N_2_ and O_2_. While NO_*x*_ is capable of destroying O_3_ in catalytic cycles in the upper and middle atmosphere, it can lead to O_3_ production in the lower atmosphere^14,21,55^. To simulate the response of the planetary atmosphere to supernovae we used a previous approach to estimate terrestrial O_3_ depletion^20^. We increased the NO production by charged particles by a factor of 100 relative to the reference simulation (which roughly corresponds to the effect of supernova exposure at 10 pc). The resulting O_3_ column density and surface UV-B are shown in Supplementary Fig. 8 by the magenta curves. We find that the supernovae impact on O_3_ column density and surface UV-B is weak, in agreement with previous work (independent of metallicity and O_2_ level). In oxygenated atmospheres (O_2_ level more than ~2%) supernovae lead to O_3_ destruction and an increase of surface UV-B. When the O_2_ content is relatively low, the additional production of O_3_ at lower altitudes in the atmosphere helps decrease the UV-B penetration (Supplementary Figs. 9 and 10 for T_eff_ of 5300 K and 6300 K, respectively).

Major volcanic eruptions can potentially damage the ozone layer^13,56^. The release of sulfur dioxide leads to the formation of sulfate particles which facilitate heterogeneous reactions that activate halogen-containing radicals that cause O_3_ destruction^57,58^. Furthermore, volcanic eruptions can directly inject halogens into the ozone layer^58,59^. To estimate potential volcanic impacts we increased the amount of sulfate particles by an order of magnitude. Reactive chlorine and bromine components were increased by injecting 3Tg HCl and 30Gg HBr per year into the lower stratosphere. This scenario mimics the end-Permian eruption of the Siberian Traps^41^. The resulting O_3_ column density and surface UV-B changes are shown in Supplementary Fig. 8 by the green curves. The O_3_-depleting effect of volcanism can be strong but weakens when the O_2_ content reaches about 10%. The volcanic impact is slightly weaker for metal-poor stars but does not drop with O_2_ content (see Supplementary Figs. 9 and 10 for T_eff_ of 5300 and 6300 K, respectively).

### Stellar spectra

The stellar spectra for different T_eff_ and metallicity values [Fe/H] have been calculated with the MPS-ATLAS code^17^. The metallicity is defined relative to the solar metallicity:

7 $$[{{{{{\rm{Fe}}}}}}/{{{{{\rm{H}}}}}}]=\,\log {({N}_{{{{{{\rm{Fe}}}}}}}/{N}_{{{{{{\rm{H}}}}}}})}_{{{{{{\rm{star}}}}}}}-\,\log {({N}_{Fe}/{N}_{{{{{{\rm{H}}}}}}})}_{\odot }$$

where N_Fe_ and N_H_ are the numbers of Fe and H atoms per volume unit. The chemical composition was taken from Asplund et al.^60^.

For each set of T_eff_ and [Fe/H] values we first computed the stellar model atmosphere using the dependence^61^ of mixing-length parameters on T_eff_ and [Fe/H] normalised to return a solar value of 1.25^62^ for solar T_eff_ and [Fe/H]. We have accounted for the line blanketing using the Opacity Distribution Functions which were computed utilising more than 100 millions of atomic and molecular lines whose spectral shape was calculated for a turbulent velocity value of 2 km s^−1^ ^17,62^. We have also accounted for the missing UV opacity following the approach by Shapiro et al.^63^.

The emergent stellar spectrum was calculated on 176 wavelength intervals exactly corresponding to our radiation code in the planetary atmospheric model (see section Atmospheric model) for 24 disk positions and then integrated over the full stellar disk. We have considered the same sources of opacity as for computing the stellar atmospheric models. In particular, a large number of spectral lines is needed for realistic calculations of the line haze which dominates the UV opacity.

Finally, the spectra have been scaled to preserve the amount of total radiative energy the planet receives. The spectra of the active Sun have been calculated following the approach by Shapiro et al.^23^ as a function of the S-index which is a standard measure of stellar activity and is proportional to the ratio of the fluxes in Ca II H and K lines and the nearby continuum^64^.

## Supplementary information

Supplementary Information

Peer Review File

## Data Availability

The data that support the findings of this study are available from the corresponding author upon request. Stellar normalized photon fluxes in the 176 spectral intervals for all metallicities and the three effective temperatures are available in source data file input_flux_photons_{T}.txt. Source data are provided with this paper. The data sets generated during the current study are available in the Edmond repository, 10.17617/3.WGVDYV. Source data are provided with this paper.

## Source data

Source Data

## Code availability

The atmospheric and MPS-ATLAS codes used in the current study are available from the corresponding author on reasonable request.
