# Supplementary material for: Metal-rich stars are less suitable for the evolution of life on their planets
Source: Nat Commun. 2023 Apr 18;14:1893. doi: 10.1038/s41467-023-37195-4 (PMC10113254; doi:10.1038/s41467-023-37195-4)
Supplement: Supplementary file 1 — Supplementary Information [file 41467_2023_37195_MOESM1_ESM.pdf]

Metal-rich stars are less suitable for the evolution of life on their planets

Supplementary information

Time, Myr ago	480<br>285<br>90	465<br>270<br>75	450<br>255<br>60	435<br>240<br>45	420<br>225<br>30	405<br>210<br>15	390<br>195<br>0	375<br>180	360<br>165	345<br>150	330<br>135	315<br>120	300<br>105
O_2, %	4<br>30<br>18	17<br>28<br>19	19<br>21<br>18	20<br>17<br>17	23<br>19<br>20	25<br>15<br>20	20<br>12<br>21	13<br>12	17<br>13	20<br>14	23<br>13	27<br>17	29<br>18
CO_2, ppm	6000<br>644<br>784	3080<br>644<br>728	2240<br>840<br>616	2940<br>1400<br>476	3640<br>616<br>364	3220<br>840<br>308	3360<br>1120<br>280	3080<br>840	840<br>756	560<br>784	420<br>1064	560<br>1400	700<br>1064

Supplementary Table 1. Evolution of O_2 and CO_2. O_2 and CO_2 on Earth from geological reconstructions.[1-3]

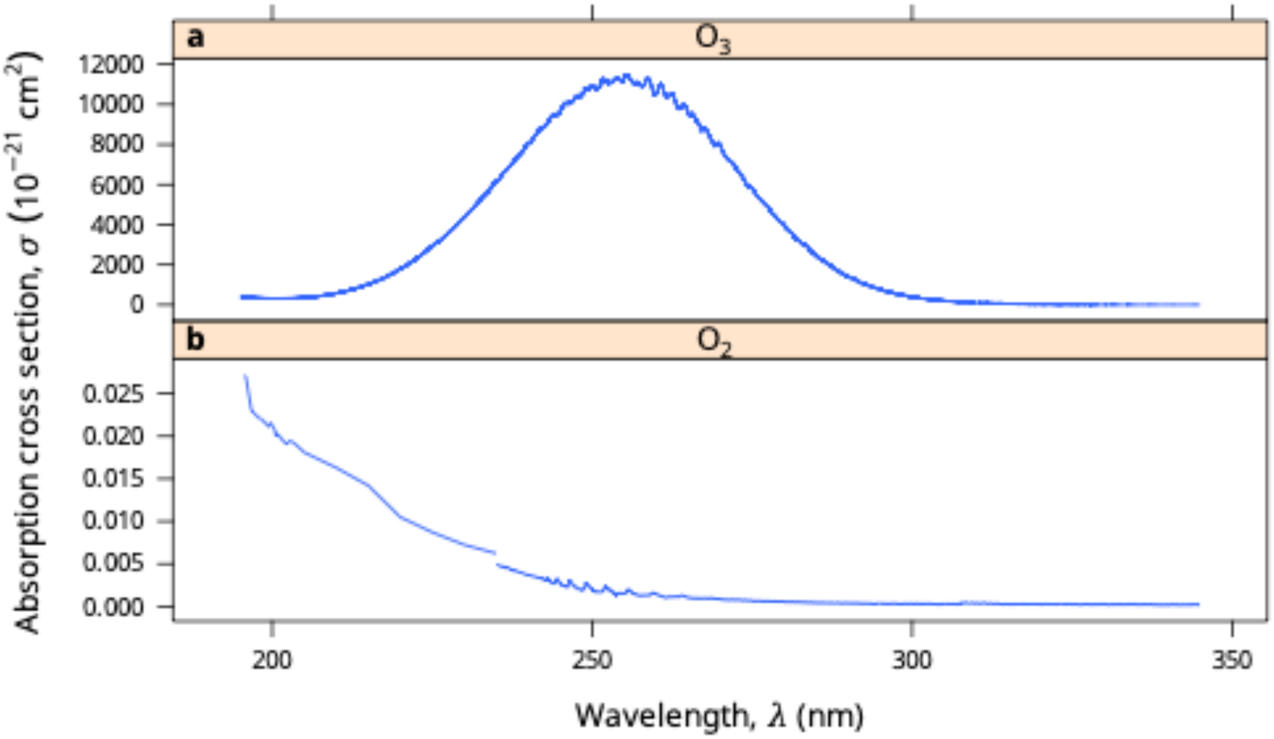

Supplementary Fig. 1. Wavelength dependent absorption cross section σ of (a) O_3 and (b) O_2 molecules. Data from Brion et al.[4] (O_3), Ogawa et al.[5] (O_2, $\lambda < 235$ nm) and Bogumil et al.[6] (O_2, $\lambda > 235$ nm), provided by the MPIC database[7]. Source data are provided as a Source Data file.

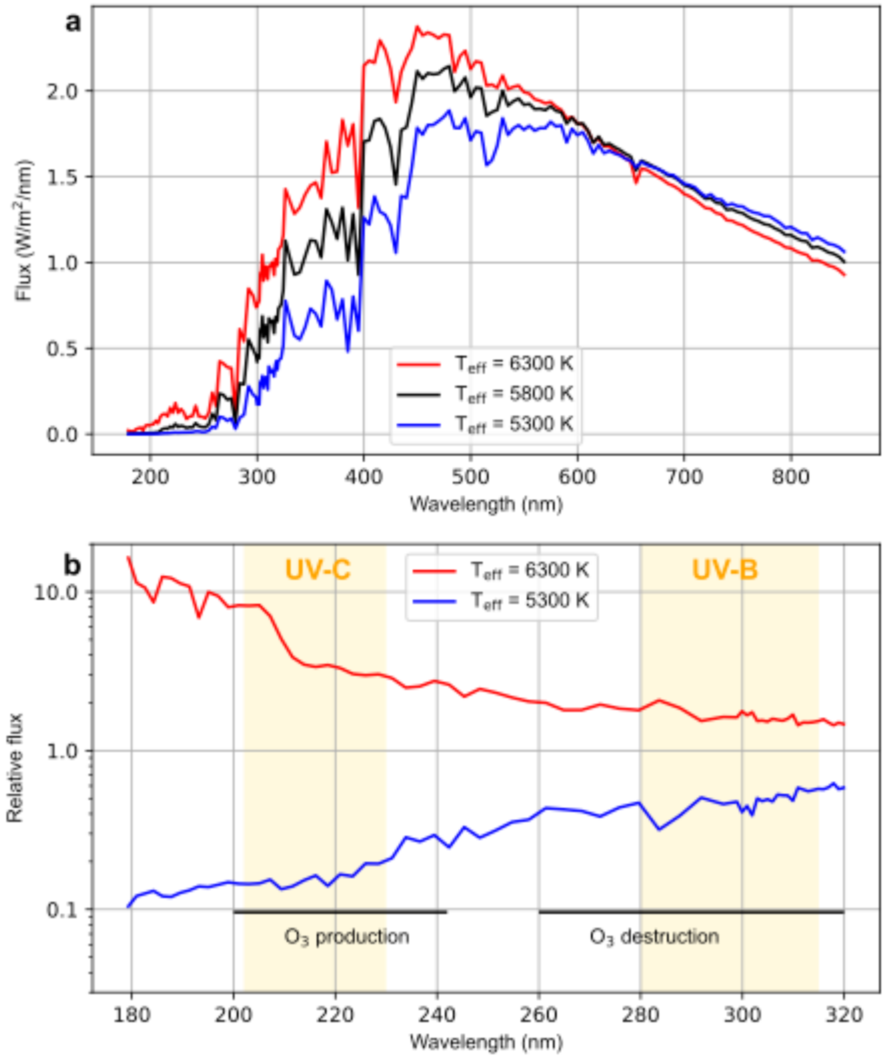

Supplementary Fig. 2. Stellar radiative spectra. a Stellar spectra calculated for solar metallicity [Fe/H] = 0 and different effective temperatures T_{eff}. The calculations were performed for a spectral range of 170 to 850 nm and the total flux normalized to the solar constant; **b** The same as **a** but the flux is shown relative to the solar flux (T_{eff} = 5800 K). Radiation in the 200 to 242 and 260 to 320 nm intervals participates in O_3 production and destruction, respectively. These spectral ranges are defined by black solid lines. The yellow shaded areas show the spectral ranges of the UV-C reaching the lower atmosphere (202 to 230 nm, left) and UV-B (280 to 315 nm, right). Source data are provided as a Source Data file.

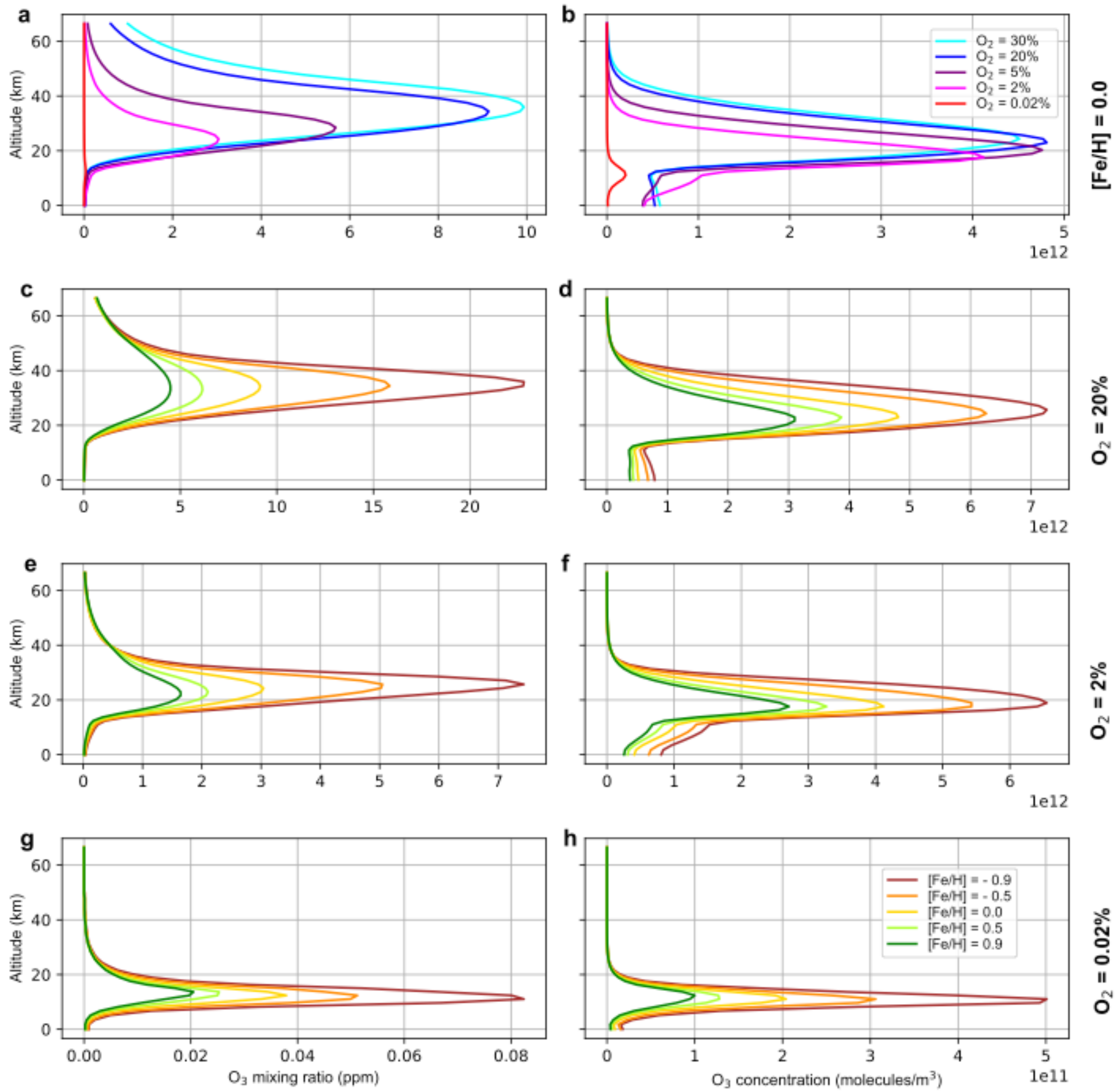

Supplementary Fig. 3. O_3 mixing ratios and concentrations. a-b, Vertical profiles of O_3 volume mixing ratios (a) and concentrations [molecules/cm^3] (b) for different O_2 contents. The calculations were performed for a host star metallicity [Fe/H] of 0.0. **c-h,** Vertical profiles of O_3 mixing ratio (c,e,g) and concentration (d,f,h) for different [Fe/H]. The calculations were performed for O_2 of 20 % (c-d), 2 % (e-f) and 0.02 % (g-h). Source data are provided as a Source Data file.

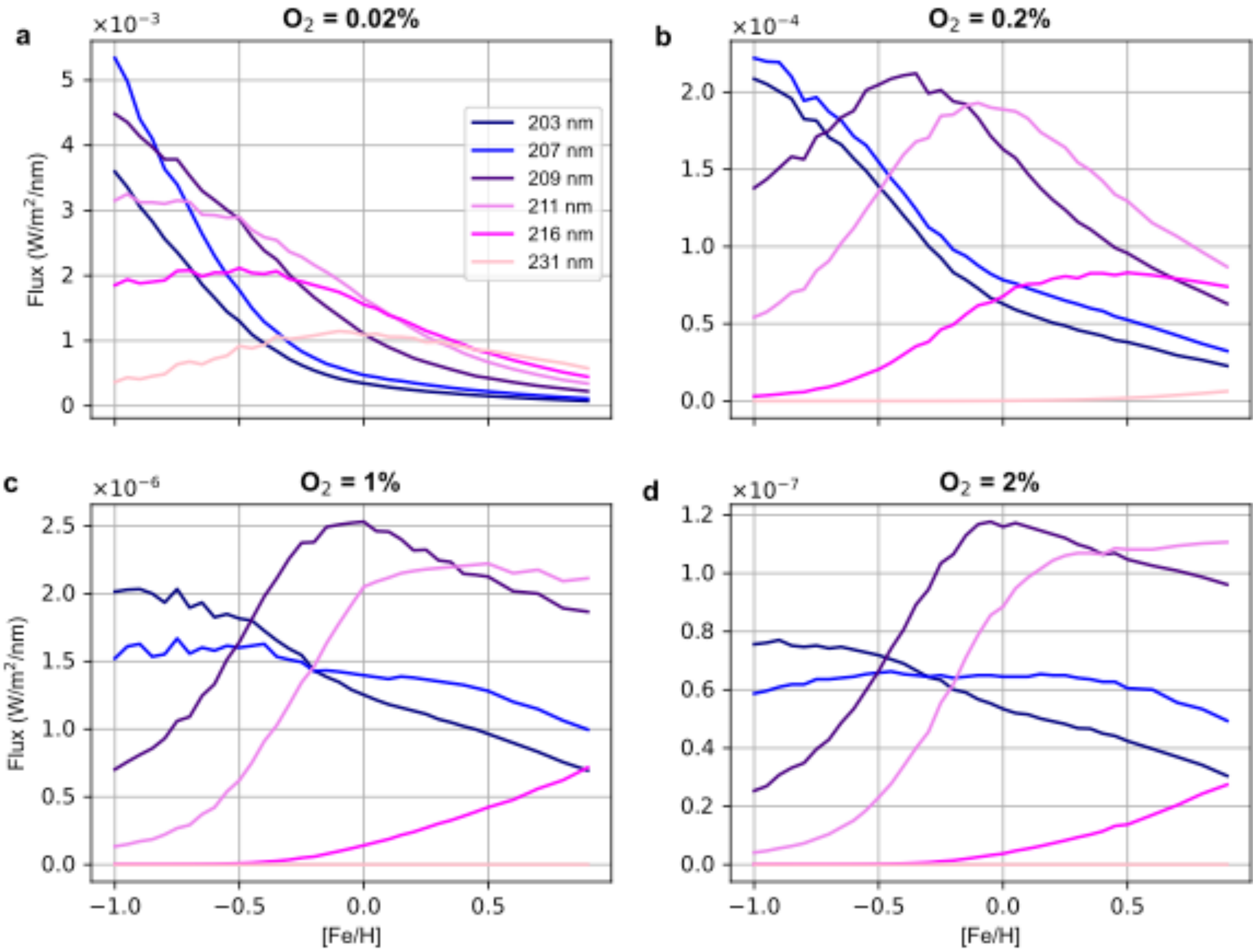

Supplementary Fig. 4. Radiative fluxes at the planetary surface. Dependencies of surface fluxes at 5800 K on [Fe/H] for O_2 levels of 0.02 % (a), 0.2 % (b), 1 % (c) and 2 % (d) calculated for selected wavelengths (203 to 231 nm). The legend presented in panels (a) is valid for all panels. Source data are provided as a Source Data file.

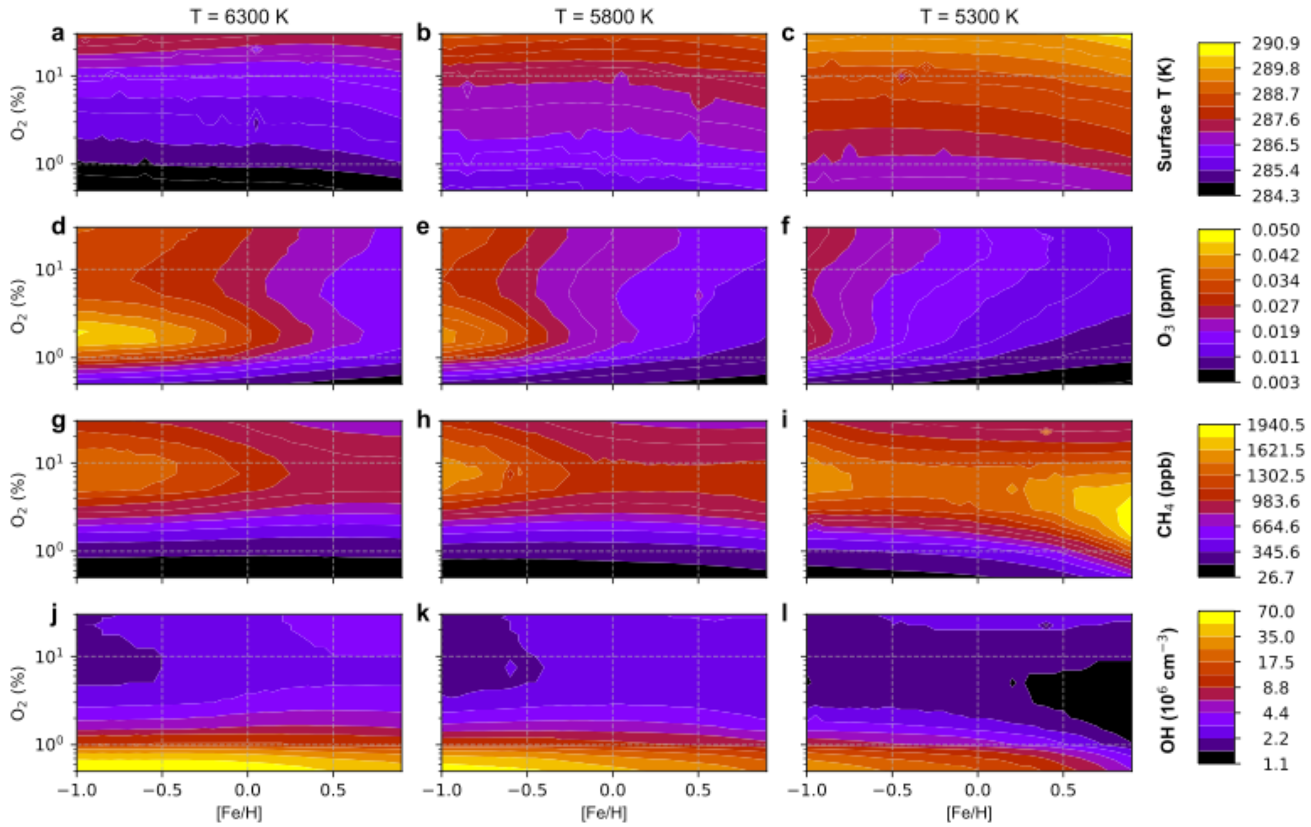

Supplementary Fig. 5. Atmospheric composition and temperature at the surface. a-o, Dependencies of the surface temperature (a–c), O_3 volume mixing ratio (d–f), CH_4 volume mixing ratio (g–i) and OH concentration (j–l) on O_2 content and metallicity [Fe/H]. The left (a,d,g,j), middle (b,e,h,k) and right (c,f,i,l) panels represent values calculated for T_{eff} of 6300 K, 5800 K and 5300 K, respectively. Source data are provided as a Source Data file.

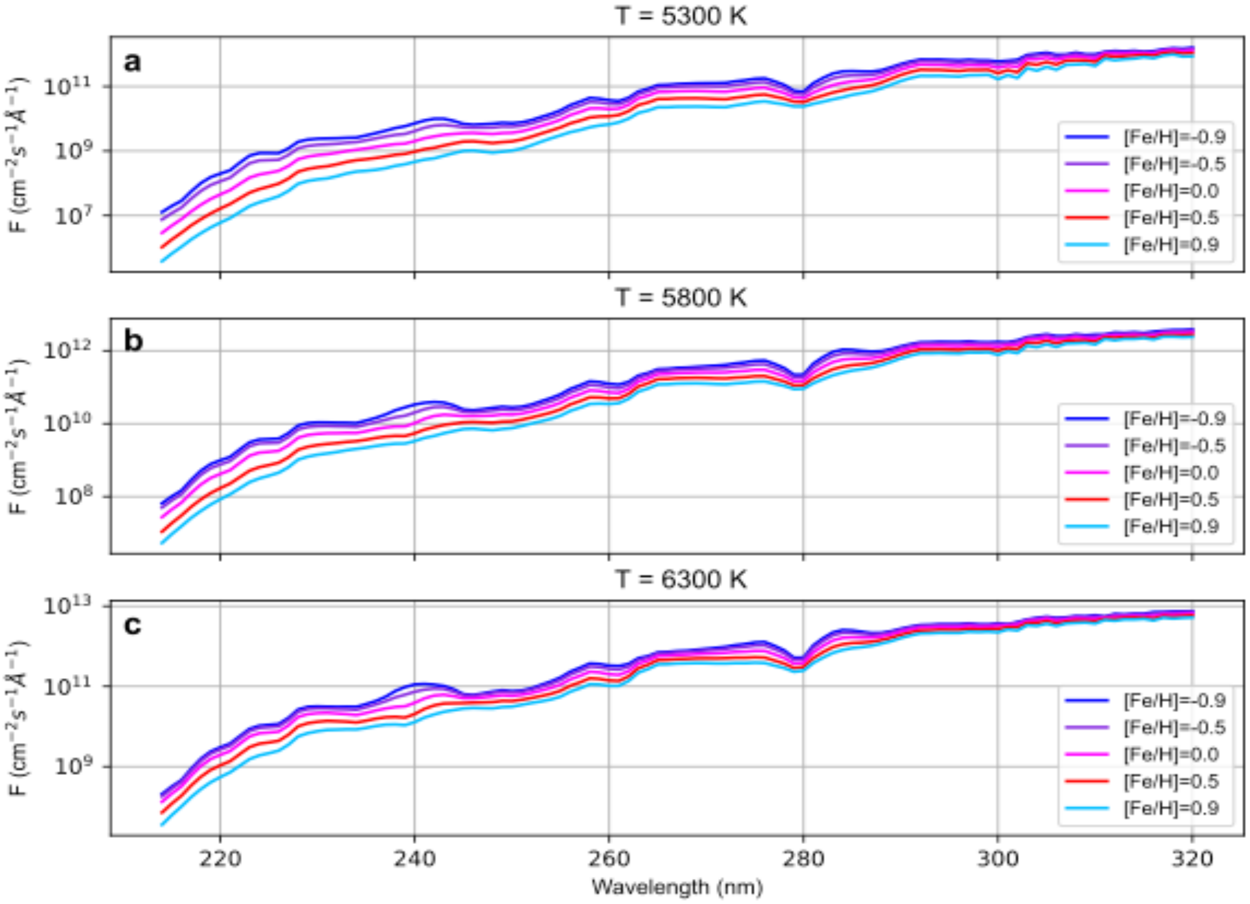

Supplementary Fig. 6. Surface actinic flux in anoxic planetary atmospheres. The spectral distributions of the surface actinic flux for different values of stellar metallicity and effective temperatures T_{eff} of 5300 (a), 5800 (b) and 6300 (c) K were obtained using the transmittance calculated by Rimmer et al.[8] for an early atmosphere composed of 80 % nitrogen and 20 % carbon dioxide. Source data are provided as a Source Data file.

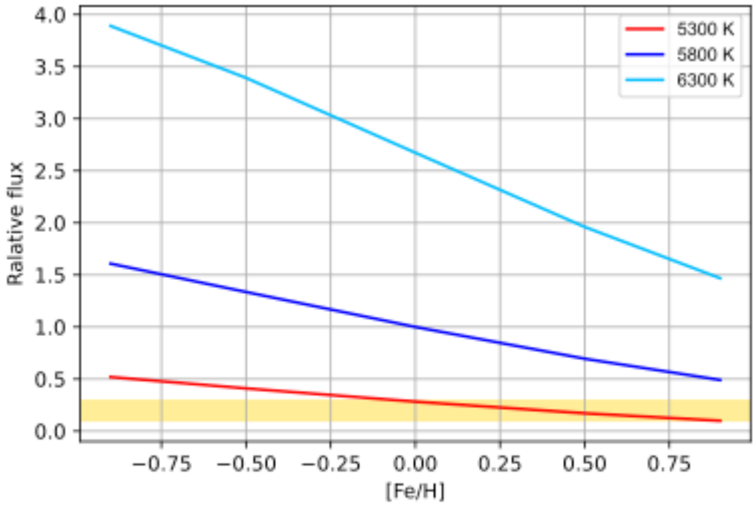

Supplementary Fig. 7. The dependence of the mean surface actinic flux on stellar metallicity. Shown are averaged over 214 to 280 nm spectral range values normalised to the value corresponding to the Sun (T_{eff} = 5800 K, [Fe/H] = 0). The yellow shaded area represents the limits of the abiogenesis zone defined by Rimmer et al.[8] . Source data are provided as a Source Data file.

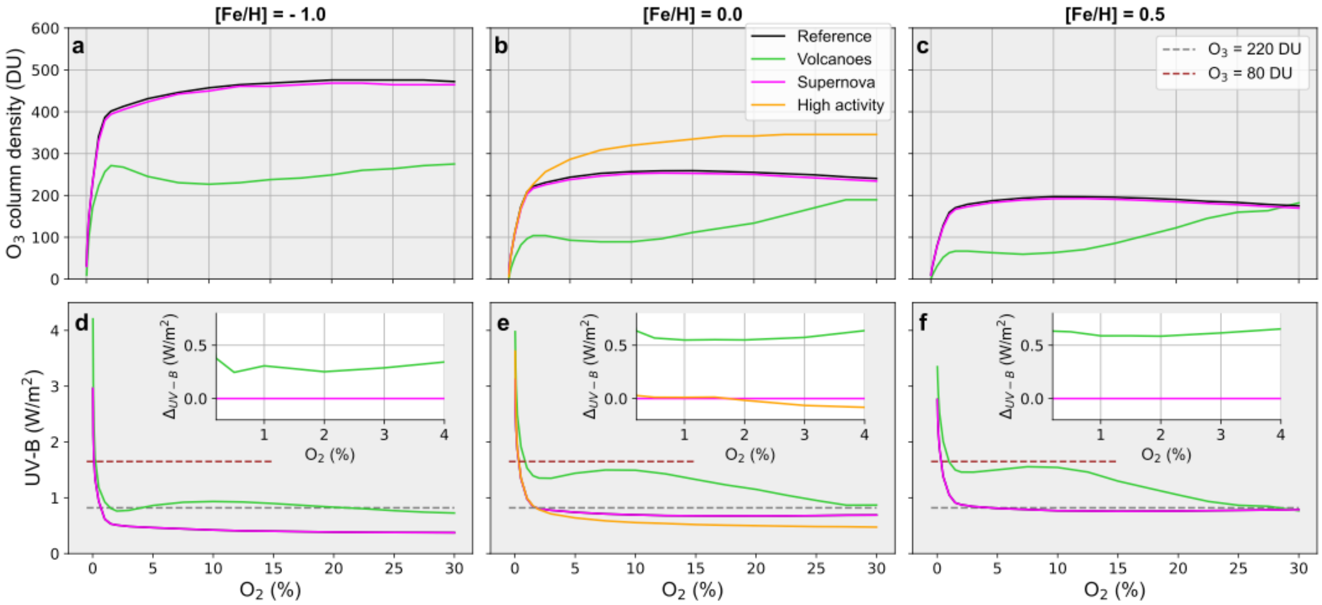

Supplementary Fig. 8. Impact of atmospheric perturbations on O_3 column density and surface UV-B. a-f, Dependencies of O_3 column density (a–c) and surface UV-B (d–f) on O_2 content obtained for a metallicity of -1.0 (a,d), 0.0 (b,e) and 0.5 (c,f). The calculations were performed for T_{eff} of 5800 K. The calculations represent strong volcanic activity (green), a supernova explosion (magenta) and high solar activity (yellow). The reference calculations are presented by the black curve. The O_3 levels of 220 and 80 DU are indicated by grey and brown dashed lines, respectively. **d–f,** The inserted plots show the differences between UV-B calculated with perturbations, e.g. strong volcanic activity (green), supernovae (magenta) and high solar activity (yellow), and obtained from the reference simulations. Source data are provided as a Source Data file.

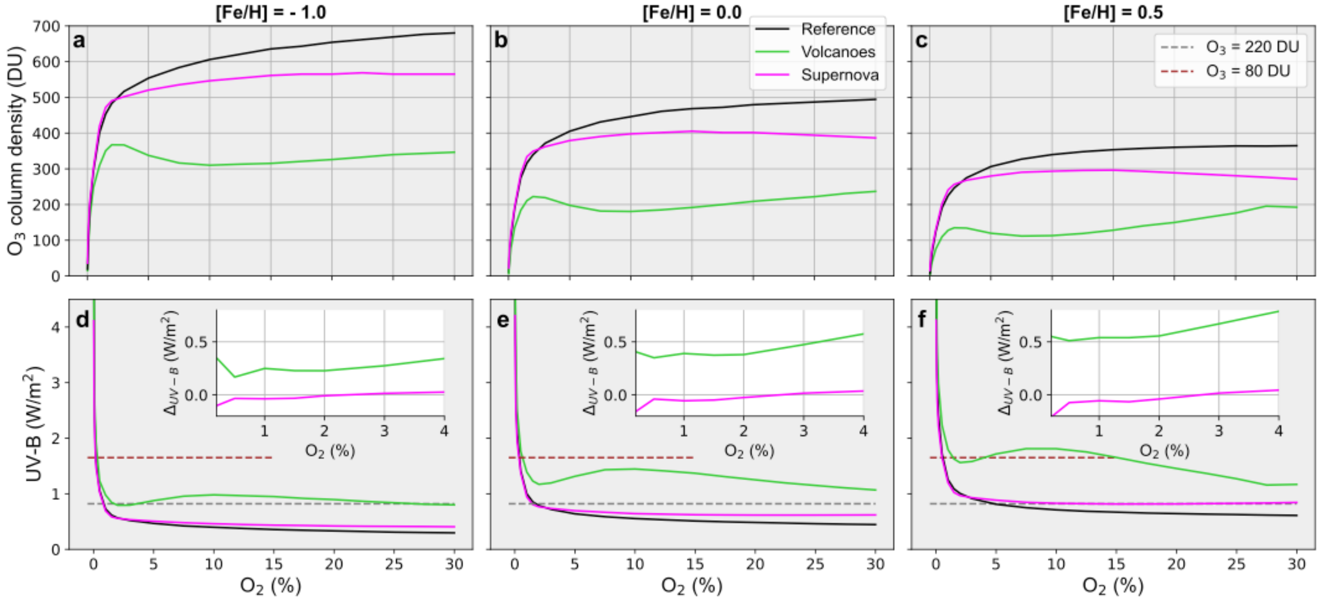

Supplementary Fig. 9. The same as Supplementary Fig. 8 but for T_{eff} of 5300 K. Source data are provided as a Source Data file.

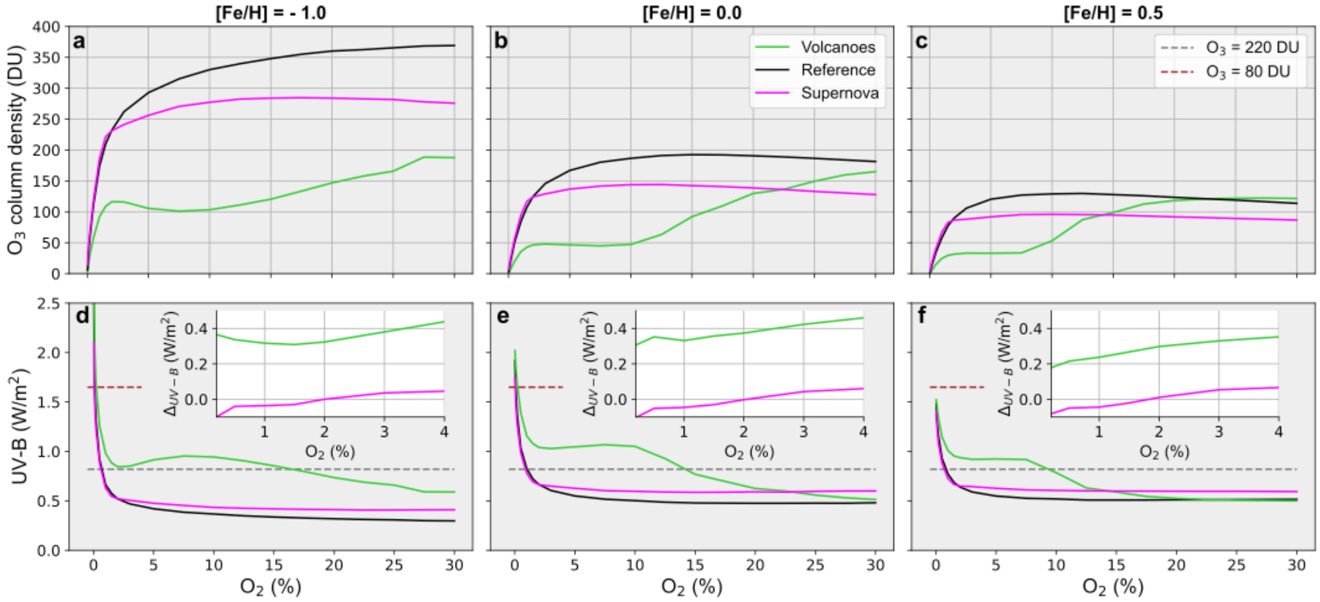

Supplementary Fig. 10. The same as Supplementary Fig. 8 but for T_{eff} of 6300 K. Source data are provided as a Source Data file.

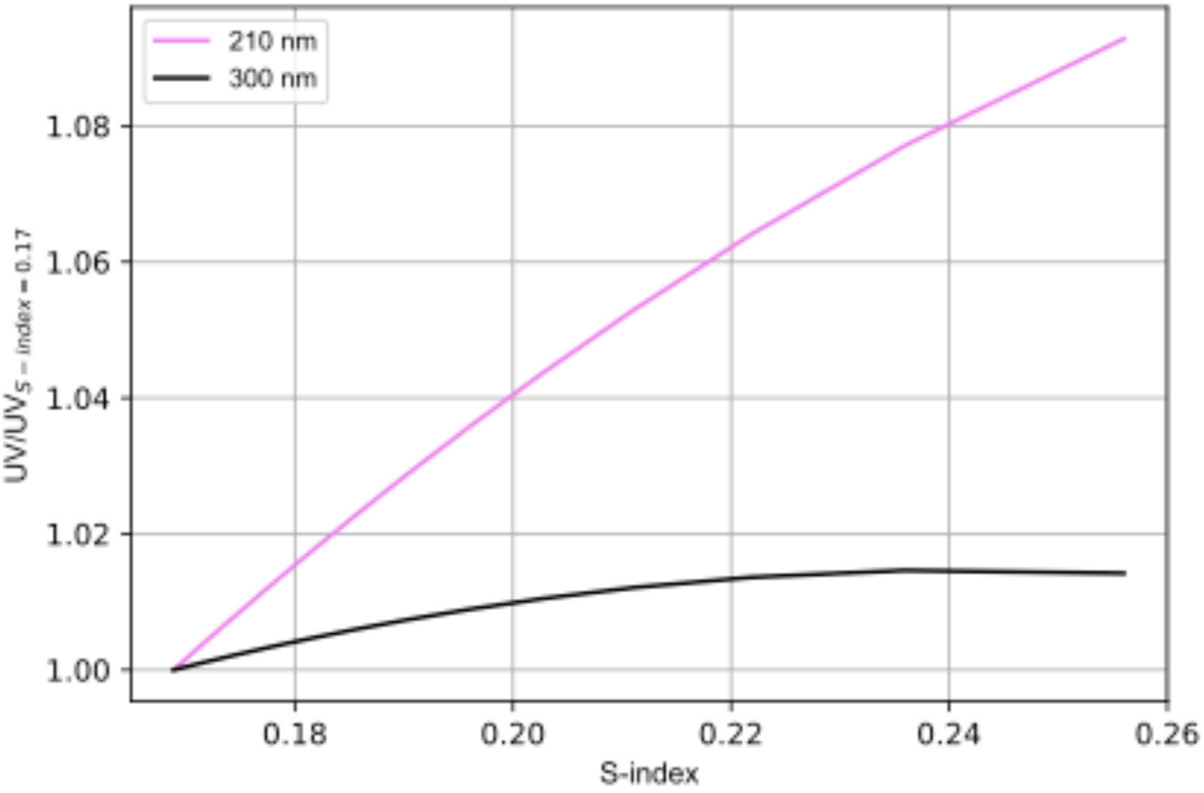

Supplementary Fig. 11. Solar space UV radiation emission changes with S-index. Changes of UV at 210 nm (magenta) and 300 nm (black) with S-index, calculated relative to the present-day value of S-index of 0.17. Source data are provided as a Source Data file.

References

1. Berner, R. A. GEOCARBSULF: A combined model for phanerozoic atmospheric O_2 and CO_2. *Geochimica et Cosmochimica Acta* **70**, 5653-5664, DOI: 10.1016/j.gca.2005.11.032 (2006).
2. Royer, D. L., Donnadieu, Y., Park, J., Kowalczyk, J. & Godderis, Y. Error analysis of CO_2.and O_2 estimates from the long-term geochemical model geocarbsulf. *Am. J. Sci.* **314**, 1259–1283, DOI:10.2475/09.2014.01] (2014).
3. Lenton, T. M. *et al.* Earliest land plants created modern levels of atmospheric oxygen. *PNAS* **113**, 9704–9709, DOI: 10.1073/pnas.1604787113 (2016).
4. Brion, J., Chakir, A., Daumont, D., Malicet, J. & Parisse, C. High-resolution laboratory absorption cross section of O_3. Temperature effect. *Chem. Phys. Lett.* **213**, 610–612, DOI: 10. 1016/0009-2614(93)89169-I (1993).
5. Ogawa, M. Absorption cross sections of O_2 and CO_2 continua in the Schumann and far-UV regions. *J. Chem. Phys.* **54**, 2550–2556, DOI: 10.1063/1.1675211 (1971).
6. Bogumil, K. *et al.* Measurements of molecular absorption spectra with the SCIAMACHY preflight model: instrument characterization and reference data for atmospheric remote-sensing in the 230–2380 nm region. *J. Photochem. Photobiol. A: Chem.* **157**, 167–184, DOI: https://doi.org/10. 1016/S1010-6030(03)00062-5 (2003).

7. Keller-Rudek, H., Moortgat, G. K., Sander, R. & Sörensen, R. The MPI-Mainz UV/VIS spectral atlas of gaseous molecules of atmospheric interest. *Earth Syst. Sci. Data* **5**, 365–373, DOI: 10.5194/ essd-5-365-2013 (2013).
8. Rimmer, P. B. *et al.* The origin of RNA precursors on exoplanets. *Sci. Adv.* **4**, eaar3302, DOI:10.1126/sciadv.aar3302 (2018)
